# The effects of career learning experiences on high school students’ engagement in learning: an explanatory sequential mixed-methods study

**DOI:** 10.3389/fpsyg.2026.1828160

**Published:** 2026-04-29

**Authors:** Xuejun Liu, Renjie Li, Qi Wu, Huan Yan

**Affiliations:** 1 Institute of Education, Xiamen University, Xiamen, China; 2 Center for Teaching and Learning Development, Xiamen University, Xiamen, China; 3 Doctoral School of Education, Faculty of Humanities and Social Sciences, University of Pécs, Pécs, Hungary; 4 School of Art and Design, Guangdong University of Technology, Guangzhou, China

**Keywords:** “3+1+2” model, career learning experience, China’s NCEE reform, learning engagement, senior high school student

## Abstract

**Introduction:**

Under China’s new National College Entrance Examination (NCEE) reform, high school students have gained greater subject choice autonomy. However, longneglected systematic career education leads to difficulties in making informed decisions, which may negatively affect learning engagement. Limited empirical research has examined the mechanism linking career learning experience to learning engagement among Chinese high school students. Drawing on Social Cognitive Career Theory (SCCT), this study investigates the predictive effect of career learning experience on learning engagement and its underlying mechanisms.

**Methods:**

A sequential explanatory mixedmethods design was adopted. Quantitatively, a convenience sample of 513 Chinese high school students completed the Career Exploration and Decision Learning Experience Scale (CEDLES) and the Utrecht Work Engagement ScaleStudent (UWESS). Data were analyzed using SPSS 26.0 (descriptive statistics, correlation analysis, stepwise regression analysis). Qualitatively, semistructured interviews were conducted with nine students and their parents, followed by thematic analysis to explain the quantitative findings.

**Results:**

Quantitative results showed that: (1) career learning experience and its dimensions (verbal persuasion, vicarious learning, positive/negative physiological and affective states, personal mastery experience) were significantly positively correlated with learning engagement and its dimensions (vigor, dedication, absorption); (2) career learning experience positively predicted learning engagement, explaining 75.9% of the variance, and its five dimensions jointly explained 76.6% of the variance. Qualitative findings revealed that enhanced selfefficacy and clarified outcome expectations were key mechanisms through which career learning experience promotes learning engagement.

**Discussion:**

This study extends SCCT to the context of China’s new NCEE and provides empirical evidence for career education interventions. The qualitative mechanism explains how career learning experience fosters learning engagement via selfefficacy and outcome expectations. It is recommended that schools implement experiential career learning activities to improve students’ subject selection competence and sustained learning engagement.

## 1 Introduction

In China, the National College Entrance Examination (NCEE) is the core education system for talent selection and cultivation, occupying a dominant position within the national talent selection framework. Since 1977, the subjects of NCEE in China have been set based on the arts–science dichotomics model, which carries drawbacks such as a single test determining one’s whole life, an excessive focus on scores, and the problem of students’ one-sided knowledge structure. In the new era where high technology drives national development, China has launched a new round of the NCEE reform recently. Compared with the traditional NCEE, the most significant change of the new NCEE is to break away from the original division between liberal arts and science tracks and implement the “3 + 1 + 2” subject selection model. “3” refers to the three national standard test subjects (i.e., Chinese, Mathematics, and Foreign Language) that are compulsory for all students; “1” refers to the preferred subject (History or Physics); and “2” refers to two of the following four elective subjects: Politics, Geography, Chemistry and Biology (Zhang, 2017). The new NCEE stimulates the vitality of ordinary high schools and provides students with multiple choices, which not only optimize students’ knowledge structure but also enhance high school students’ learning motivation. Besides, it fully reflects the respect for students’ dominant status and growth differences, highlighting the value orientation of “student-centered.” However, the granting of rights is not equal to the acquisition of ability. For a long time, China’s career education has not received due attention. The absence of career education has made it difficult for high school students to adapt to the subject selection system of the new NCEE reform, resulting in a situation where they possess the right to choose but lack the ability to make informed decisions (Zhang et al., 2024; Gu et al., 2020). More specifically, as they lack an adequate understanding of universities and majors, they often encounter confusion, ambiguity, and even misconceptions when making their choices. Consequently, they often feel confused and anxious when faced with multiple choices, and blindly make their selections (Zhu et al., 2024).

In addition, the current surveys showed that Chinese high school students face greater exam pressure, many subjects to learn, difficult knowledge, and other problems, resulting in their distraction on learning, absence of perceived value and meaning in learning, and generally low level of learning engagement (Zhao et al., 2015; Oubibi et al., 2023). As Chinese schools undergo major reforms and continue to emphasize quality education, it is important to enhance students’ engagement in the learning process (Yu et al., 2019). Besides, achieving personal happiness and life fulfillment through learning is a critical issue for China and even the world (Yu et al., 2018; Sever et al., 2014). Thus, researches on high school students’ learning engagement play a crucial role in alleviating their academic burnout, enhancing their academic performance, and improving their core competencies.

This study, based on conceptual analysis and SCCT theoretical analysis, incorporates career learning experience and learning engagement into the same research framework and holds that more career learning experience can improve high school students’ efficacy in individual subject and major selection, enabling them to make reasonable decisions on subject and major, to promote their learning motivation, learning interest and learning investment, and thus achieve the “student-centered” value orientation of the new NCEE (Pang et al., 2025). At the same time, for education reform and school intervention, it is necessary to carry out targeted empirical research to accumulate evidence to deeply understand the influence mechanism of high school students’ learning engagement. Therefore, in the context of the new NCEE reform in China, this study takes Chinese high school students as samples to investigate the effect of career learning experience on high school students’ learning engagement and the mechanism between them.

## 2 Literature review and research hypothesis

### 2.1 Research hypothesis

From the perspective of conceptual definition, career learning experience is the source of career efficacy, in other words, it is an important antecedent variable of career efficacy, and it can have a direct impact on career-related efficacy, such as career decision-making self-efficacy and career efficacy (Ireland, 2017). Previous studies have shown that career-related efficacy can significantly positively predict individual learning engagement level (Jiang et al., 2022). Therefore, from this view, this study hypothesized that career learning experience can significantly predict the learning engagement level of high school students.

From a theoretical point of view, career learning experience is an important information source for career self-efficacy. In 1994, Lent, Brown, and Hackett proposed social cognitive career theory (SCCT) based on Bandura’s social learning theory. According to the SCCT theoretical model, career learning experience can interact with individual characteristics and background environment to have a direct impact on self-efficacy and outcome expectation, that is, career learning experience can affect individuals’ self-efficacy and outcome expectations, and thus affect their interest and effort in achieve personal goals. Moreover, previous studies have shown that interest, self-efficacy, and outcome expectations are important antecedent variables of individual learning engagement (Sheu and Bordon, 2017; Turner et al., 2019). Therefore, under SCCT, career learning experience can indirectly enhance the level of learning engagement by improving learning interest, self-efficacy, learning goals, outcome expectations (Liu et al., 2024). The underlying mechanisms of action are complex and diverse. It is difficult to conduct extensive and comprehensive mechanism exploration from a variable perspective. This study hypothesizes that career learning experiences can enhance the level of learning engagement through certain influence mechanisms, and intends to explore these influence mechanisms through qualitative text analysis.

Previous studies mainly focused on the relationship between learning engagement and factors such as parenting style, teacher-student relationship, intrinsic motivation, academic emotion, and self-efficacy, while neglecting to explore the connection between certain learning experiences and learning engagement. However, researchers have found that high-impact research learning experiences can affect individual learning engagement (Miller et al., 2011). Therefore, in the context of a new round of NCEE reform in China, based on the literature review, this study proposed the hypothesis that career learning experience can directly affect the learning engagement of high school students, and indirectly affect high school students’ learning engagement through certain mechanisms. This study investigates the impact of career learning experiences on high school students’ learning engagement and its underlying mechanisms, using a sample of Chinese high school students.

### 2.2 Career learning experiences

Career learning experiences (CLE), namely, source information of self-efficacy related to career exploration and decision-making, include personal mastery experience (PME), verbal persuasion (VP), vicarious learning (VL), physiological and affective states (PAS) (Ireland and Lent, 2018). PME represents an individual’s successful experiences in career exploration and decision-making. VP represents the affirmation of an individual’s successful performance from someone they trust when they are exploring careers and making decisions. VL represents the experience an individual gains by observing a role model who successfully chooses an major or a career path. PAS represents an individual’s positive and negative emotion states during career exploration and decision-making (Lent et al., 1994; Ireland and Lent, 2018).

Studies have shown that factors that affect the career learning experience mainly include personal characteristics and growth background. Personal characteristics include personality, gender, race and other factors. For example, an individual characterized by conscientiousness, one of the Big Five personality traits, has more learning experiences in conventional and social career fields (Schaub, 2003). Gender and racial identity can influence an individual’s career learning experience (Lannin et al., 2023). Growth background, which is an influencing factor of people’s career choice behavior can be divided into long-term growth background and short-term environment. Compared with short-term environment, long-term growth background-which involves parents’ parenting style, family economic status, teacher-student relationship, and school atmosphere has a greater impact on individual career learning experience. For example, Thompson and Dahling (2012) conducted a controlled study based on participants’ gender, and the results showed that socioeconomic status can significantly affect individuals’ career learning experiences in research, business, and conventional professions.

Career learning experience can not only directly affect self-efficacy and outcome expectations in Holland’s six personality types and their corresponding career paths, but also influence outcome expectations through self-efficacy (Williams, 2010). Learning experience plays a mediating role between parental support, self-efficacy, and outcome expectations (Garriott et al., 2014). Besides, career learning experience can play a mediating role between person-environment fit and individual career development, such as career interest, career choice, career action, and so on. Career learning experience helps to mediate the relations of personality and social support to self-efficacy and outcome expectations, as well as to goals and career decisions (Ireland and Lent, 2018).

### 2.3 Learning engagement

In the early 20th century, under the trend of positive psychology, work engagement became a research focus. With the deepening of research on work engagement, researchers found that the phenomenon of engagement also existed in the student group, and they then started to focus on learning engagement (Schaufeli et al., 2002). Among different definitions of learning engagement by many scholars, Schaufeli et al. (2002) view has been widely recognized because the definition he proposed remained stable even across cultural contexts, and he has developed corresponding measurement tools and applied them in empirical studies. Based on the above, this study adopted the definition proposed by Schaufeli, defining learning engagement (LE) as students’ active and concentrated state during learning, including vigor (VI), dedication (DE), and absorption(AB). VI refers to finding it difficult to detach oneself from learning, being willing to work hard in learning, and persevering even when encountering difficulties. DE refers to being fully engaged in learning and feeling the significance and challenge of learning. AB refers to being completely absorbed in learning, feeling that time passes quickly, and having the difficulty to separate oneself from learning.

Learning engagement can have a significant positive effect on students’ attitudes and behaviors in the school environment, including students’ learning interests, learning performance, academic achievement, physical health, and satisfaction (Chan and Ko, 2019; Ying et al., 2023). In addition, learning engagement can also predict an individual’s enrollment, dropout, and career development 10 years later (Orthner et al., 2010). Therefore, exploring the factors influencing learning engagement is particularly crucial for promoting the development of students.

Previous studies have found that students’ learning engagement could be influenced by individual characteristics, family factors, and school factors. In terms of individual characteristics, age, commitment, adaptive perfectionism, cognitive need and cognitive motivation, learning motivation, learning interest, self-efficacy, and future orientation can significantly positively predict students’ learning engagement level (Efklides, 2017; Efklides et al., 2017; Ramirez-Arellano et al., 2018). In terms of family factors, it has been confirmed that family support, family socioeconomic status, family environment, family characteristics, parenting style, parental involvement in education, parental expectations, and other factors have an impact on students’ learning engagement level (Oubibi et al., 2023; Tong et al., 2024). In terms of school factors, some research have revealed that teacher support, teacher-student relationship, peer communication, school atmosphere, and other factors significantly affect students’ learning engagement level (Acosta-Gonzaga and Ramirez-Arellano, 2022; An et al., 2022).

## 3 Research design

Grounded in the pragmatic paradigm as its foundation of legitimacy, mixed methods research can integrate diverse forms of data and combine the strengths of different analytical approaches. This enables it to provide richer, deeper insights and understanding for effectively addressing research questions (Toyon, 2021). The current study employed the a sequential explanatory mixed-methods design, specifically using quantitative research methods first to conduct empirical analysis of complex educational phenomena, followed by qualitative research methods to explore the underlying mechanisms behind the quantitative findings.

### 3.1 Participants

This study was conducted in two high schools from City D, China. As high school students are minors and has little time to go online due to academic pressure, this study adopted the approach of contacting class teachers to uniformly distribute questionnaires. In the quantitative research stage, by using a convenience sampling method, a total of 557 questionnaires were issued and collected. After excluding invalid responses due to patterned answering, excessively short or long completion times, or failure to pass attention check questions, 513 valid questionnaires were obtained, yielding an effective response rate of 92.101%. This sample size surpasses the 5:1 parameter-to-respondent ratio recommended for SEM (Bentler and Chou, 1987), ensuring statistical robustness. In terms of gender, there were 214 boys and 299 girls in the valid sample. In terms of grade, there were 181 students in the 10th grade, 108 students in the 11th grade, and 224 students in the 12th grade.

In the qualitative research stage, we recruited 9 high school students and their parents through a combination of online recruitment and offline promotion. The basic information of the interview samples is shown in Table 1. Among them, the gender ratio of students was relatively balanced, and the grade distribution was even.

**Table 1:** Basic information of interview samples.

Classes	Number	Gender	Grade/degree	Status/occupation
Student	A1	Male	Grade 1	Student
	A2	Female	Grade 1	Student
	A3	Female	Grade 2	Student
	A4	Female	Grade 1	Student
	A5	Male	Grade 2	Student
	A6	Male	Grade 3	Student
	A7	Female	Grade 3	Student
	A8	Female	Grade 2	Student
	A9	Male	Grade 1	Student
Parents	B1	Female	Undergraduate degree	Industrialists and businessmen
	B2	Female	Junior school degree	Unemployed
	B3	Male	Undergraduate degree	State-owned enterprise staff
	B4	Female	Junior school degree	Migrant worker
	B5	Female	High school degree	Unemployed
	B6	Female	Junior school degree	Migrant worker
	B7	Female	Junior school degree	Migrant worker
	B8	Female	Primary school degree	Migrant worker
	B9	Female	Junior school degree	Migrant worker

### 3.2 Instruments

#### 3.2.1 Career exploration and decision learning experience scale

Currently, the “Career Exploration and Decision Learning Experience Scale (CEDLES)” is an internationally recognized and widely applied measurement tool for career learning experiences, demonstrating high validity. This study employed the revised Chinese version of the CEDLES developed by Zhou and Xu (2022) to measure high school students’ career learning experience levels. The scale included five dimensions, examining students’ personal mastery experience (PME), verbal persuasion (VP), vicarious learning (VL), positive physiological and affective states (PPAS), and negative physiological and affective states (NPAS). There were 20 items in total with a five-point Likert-type scale, ranging from “1” for “strongly disagree” to “5” for “strongly agree.” The higher the score, the higher the level of career learning experience among high school students. In this study, the Cronbach’s *α* coefficient of the scale was 0.861, and the Cronbach’s *α* coefficients for the sub-dimensions of VP, PPAS, NPAS, VL, and PME were 0.855, 0.878, 0.839, 0.864 and 0.860, respectively. All of these demonstrated that the scale had good reliability. Besides, the results of the validated factor analysis showed a good fit: $\chi^{2}$ /*df* = 1.490, CFI = 0.988, TLI = 0.986, GFI = 0.957, SRMR = 0.028, RMSEA = 0.031, confirming the good validity of this scale.

#### 3.2.2 Utrecht work engagement scale-student

Based on the definition of learning engagement, Schaufeli developed the “Utrecht Work Engagement Scale-Student” (UWES-S), which has gained widespread application and demonstrated high validity (Schaufeli et al., 2002). This study employed the Chinese version of the UWES-S, translated and revised by Fang et al. (2008), to measure learning engagement levels among high school students. The Chinese version of UWES-S includes three dimensions, examining students’ vigor (VI), dedication (DE), and absorption (AB). There are 17 items in total with a seven-point Likert-type scale, ranging from “1” for “never” to “7” for “always/every day.” The higher the score, the higher the level of students’ learning engagement. In this study, the Cronbach’s *α* coefficient of this scale was 0.969, and the Cronbach’s α coefficients for the sub-dimensions of VI, DE and AB were 0.925, 0.900 and 0.922, respectively. All of these demonstrated that the scale had good reliability. Moreover, the results of the confirmatory factor analysis showed a good fit: $\chi^{2}$ /*df* = 1.581, CFI = 0.991, TLI = 0.989, GFI = 0.960, SRMR = 0.046, RMSEA = 0.034, the good validity of the scale.

### 3.3 Procedures

This study was conducted using the following steps. First, we distributed questionnaires, which included basic information, the CEDLES, and the UWES-S, to students of two high schools to test the research hypothesis that career learning experiences have an impact on students’ learning engagement. Secondly, we conducted semi-structured interviews to get more specific information. Each pair consisting of a recruited high school student and their parent was grouped for a joint interview. The interviews were conducted via online video calls, and each lasted approximately 40 min. Before the interview began, the interviewees were informed of the purpose of the interview and the significance of the research. To ensure complete documentation of the interview content and facilitate subsequent textual analysis, the entire interview was audio-recorded after obtaining the consent of the interviewees. Notably, based on the questions in the interview outline, the interviews were carried out flexibly according to the status and interest points of the interviewees, to guide the interviewees to express their own opinions more frequently. Thirdly, the results of questionnaires were analyzed. Then, we employed content analysis to examine the interview transcripts, and used these results to interpret the questionnaire findings, and construct a model illustrating the influence mechanism of career learning experiences on high school students’ learning engagement.

### 3.4 Date analysis

In the quantitative analysis phase, SPSS 26 software was employed to conduct data analysis on the valid questionnaires to test the research hypotheses. In the qualitative analysis phase, after each interview group concluded, the interview materials were sorted out in a timely manner, and the recordings were transcribed verbatim, ultimately yielding over 20,000 words of interview transcripts. Using Nvivo 11 software as an auxiliary tool for qualitative research, seven sets of materials were coded at three levels: open coding, axial coding, and selective coding. Finally, based on these codes, we constructed a model illustrating the influence mechanism of career learning experiences on high school students’ learning engagement. However, with the purpose of saturation testing, the remaining 2 groups of materials were re-coded according to the above coding process. The results indicated no emergence of new conceptual categories and the logical relationship between the existing categories was consistent with the above research results. Therefore, the data can be considered saturated, and the model construction is deemed valid.

## 4 Results

### 4.1 The quantitative analysis

As shown in Table 2, the overall mean of career learning experience was 3.524 (above the scale midpoint of 3), suggesting a moderately high level. Among its dimensions, verbal persuasion (VP) scored the highest (M = 3.606), followed by vicarious learning (VL) (M = 3.603), positive physiological and affective states (PPAS) and personal mastery experience (PME) (M = 3.560 for both), while negative physiological and affective states (NPAS) scored the lowest (M = 3.291). For learning engagement, the overall mean was 4.637 (above the midpoint of 4), also indicating a moderately high level. Dedication (DE) had the highest mean (M = 4.699), followed by absorption (AB) (M = 4.611), and vigor (VI) the lowest (M = 4.601).

**Table 2:** Descriptive statistics of career learning experience and learning engagement (*N* = 513).

Dimension/variable	No. of items	M	SD
Verbal persuasion (VP)	4	3.606	0.959
Negative physiological and affective states (NPAS)	4	3.291	1.059
Positive physiological and affective states (PPAS)	4	3.560	0.909
Vicarious learning (VL)	4	3.603	0.956
Personal mastery experience (PME)	4	3.560	0.945
Career learning experience (total)	20	3.524	0.828
Vigor (VI)	6	4.601	1.439
Dedication (DE)	5	4.699	1.351
Absorption (AB)	6	4.611	1.409
Learning engagement (total)	17	4.637	1.346

Pearson product–moment correlation analyses were performed to assess the correlations among career learning experience, learning engagement, and their sub-dimensions. As shown in Tables 3, 4, career learning experience was significantly positively correlated with learning engagement, and all sub-dimensions of the two variables were also significantly positively correlated with each other.

**Table 3:** Correlation analysis results of career learning experience and learning engagement.

Variable	CLE	LE
CLE	1	
LE	0.872**	1

*N* = 513, *p < 0.05, **p < 0.01, ***p < 0.001; CLE, career learning experience; LE, learning engagement.

**Table 4:** Correlation analysis results of each variable dimension.

Variable	VP	NPAS	PPAS	VL	PME	VI	DE	AB
VP	1							
NPAS	0.511^**^	1						
PPAS	0.764^**^	0.498^**^	1					
VL	0.794^**^	0.478^**^	0.752^**^	1				
PME	0.823^**^	0.533^**^	0.796^**^	0.827^**^	1			
VI	0.754^**^	0.560^**^	0.725^**^	0.757^**^	0.786^**^	1		
DE	0.790^**^	0.537^**^	0.741^**^	0.787^**^	0.806^**^	0.878^**^	1	
AB	0.757^**^	0.540^**^	0.735^**^	0.774^**^	0.795^**^	0.893^**^	0.889^**^	1

*N* = 513, *p < 0.05, **p < 0.01, ***p < 0.001; VP, verbal persuasion; NPAS, negative physiological and affective states; PPAS, positive physiological and affective states; VL, vicarious learning; PME, Personal mastery experience; VI, vigor; DE, dedication; AB, Absorption.

Multiple regression analyses were conducted to examine the predictive effect of career learning experience on learning engagement. First, a stepwise regression analysis was performed with career learning experience as the independent variable and learning engagement as the dependent variable. As shown in Table 5, the collinearity diagnostics indicated that tolerance (TOL) values were greater than 0.2 and variance inflation factor (VIF) values were less than 10, suggesting that multicollinearity was not a concern among the independent variables. Career learning experience entered the regression equation and explained 75.9% of the variance in learning engagement. This result indicates that career learning experience significantly and positively predicts learning engagement. Second, a stepwise regression analysis was conducted with verbal persuasion (VP), negative physiological and affective states (NPAS), positive physiological and affective states (PPAS), vicarious learning (VL), and personal mastery experience (PME) as independent variables, and learning engagement as the dependent variable. As shown in Table 6, the collinearity diagnostics revealed that all tolerance (TOL) values were greater than 0.2 and all variance inflation factor (VIF) values were less than 10, indicating no multicollinearity issues. All five dimensions—VP, NPAS, PPAS, VL, and PME—entered the regression equation and together explained 76.6% of the variance in learning engagement. This result demonstrates that each dimension of career learning experience (i.e., VP, NPAS, PPAS, VL, and PME) significantly and positively predicts learning engagement.

**Table 5:** Regression analysis results of career learning experience and learning engagement.

Variable	Dimension	*R*^2^	*β*	*t*	TOL	VIF
LE	CLE	0.759	0.872	40.176***	1.000	1.000

*N* = 513, *p < 0.05, **p < 0.01, ***p < 0.001; LE, learning engagement; CLE, Career learning experience.

**Table 6:** Regression analysis results of all sub-dimensions of career learning experience and learning engagement.

Variable	Dimension	*R*^2^	*β*	*t*	TOL	VIF
LE	VP	0.766	0.401	6.021***	0.210	4.773
	NPAS		0.352	6.045***	0.267	3.745
	PPAS		0.278	4.745***	0.263	3.802
	VL		0.166	5.082***	0.692	1.444
	PME		0.199	3.531***	0.315	3.176

*N* = 513, *p < 0.05, **p < 0.01, ***p < 0.001; LE, learning engagement; VP, verbal persuasion; NPAS, negative physiological and affective states; PPAS, positive physiological and affective states; VL, vicarious learning; PME, personal mastery experience.

### 4.2 The qualitative analysis

During the open coding phase, seven groups of text materials were summarized sentence by sentence to categorize the content. Similar categories were consolidated, while non-representative concepts were discarded. Finally, a total of 77 reference points were sorted out as concepts, and 15 nodes were named as categories. Selected representative original statements and open coding results are presented in Table 7.

**Table 7:** Open coding result.

Categories	Original representative statements
Information collection	B5: We are not so clear, for example, if you like physical chemistry so much, what kind of industry you can choose in the future, what kind of work you can do. All of these are unclear for us because we do not have many channels to know. In addition to those that can be found on the Internet, I hope the school can provide some channels so that we can study more actively with these information. (Expand information channels)
Understanding of the environment	A5: Let us know what other majors are available. If we do not know these, it is still difficult to choose a major. First of all, we need to know what majors we can choose. Now we are learning specific knowledge such as physical chemistry. However, it’s essential to tell us what kind of college majors we can take in these subjects, how many scores are required for these majors, or which university offers better majors. After understanding these, we would study with more specific goals. (Understand the external professional and professional environment)
Self-assessment	A1: I know that there is an assessment for students, which involves an interview where they ask a series of questions. Afterward, they provide an evaluation outlining the student’s general tendencies, essentially determining what personality traits suit them best, and then make a matching recommendation. This helps the students understand what they are truly suited for. I think it is excellent. I (Know yourself)
Self-reflection	A7: The role of a career model is that make me feel that if others are outstanding, I should not let myself fall too far behind. IT prompts me to fosters a sense of comparison, and serves as a motivating force, encouraging me to study diligently. (Reflect on oneself)
Learning interest	A2: We have a class dedicated to exploring interests. In that class, we have six interest-related islands to choose from. This helps us learn about our career interests, and then combine our career interests with the subjects we learn, which can arouse our learning interests. (Stimulate learning interest)
Learning motivation	B1: Whether a student performs well academically depends entirely on how hard the parents work. If parents put in the effort, the student’s grades improve; if parents slack off, the student’s grades drop immediately. As soon as I do not have time to pay attention to him, his grades drop, so it becomes entirely parent-driven learning, which is not right. I am joking now even before. I said I hope he could find a girlfriend who had good grades, and then he may be motivated. I think the role of career lessons or career experiences for them is helping them set a goal, then they would generate learning motivation. Once they has such a career plan and their own objectives, they do not need us to push them at all because they think that they should learn well. (Stimulate learning motivation)
Target selection	B2: Today’s parents give too much to their children, and children receive too much without learning to release. Unable to release, they cannot make choices. They lack independence, judgment, self-understanding, and social skills. Career courses or experiential career activities can help children develop the ability to make autonomous choices. (Cultivate the ability to choose)
Making a plan	A6: At the end of the class, the teacher asked us to write a short essay outlining our plans and goals. So we need to write our ideas down on the paper rather than just keeping them in our heads, and this can help me study more systematically and make it easier to achieve my objectives. (Make a plan)
Problem solving	A7: The students struggle academically need extra encouragement. We should support them to participate in career activities. I think this process is very important. The journey of striving toward a goal is what truly matters and the outcome itself may not be crucial because sometimes we cannot achieve the goals. However, this process of hard work must be a very important experience or a precious treasure in life. When future setbacks arise, including academic challenges, she will seek solutions rather than retreat, striving to rise above them. (Cultivate students’ problem-solving ability and positive attitude)
Logical thinking	B3: I should pay attention to both life and study, but in fact, sometimes it is a kind of over-nutrition. She has too little personal space and room for self-expression. Therefore, I hope to make some improvements through high school career classes. I’m trying to give her more freedom, and you can feel that she actually has her own thinking logic from her answers, but she still needs to practice more. I hope that career-related knowledge and problem-solving methods can train her expression and logical thinking, which will definitely be helpful to her daily learning. (Exercise logical thinking)
Parent-adolescent communication	B7: We, as parents, strongly support her to participate in some career activities and learn about career planning. Our support can also give her some emotional encouragement. A positive attitude and a supportive family atmosphere undoubtedly contribute to her academic success. (parent–child relationship)
Peer communication	B2: I feel that I really need to communicate more with friends and role models. With everyone’s help, I can gain clearer perspectives on certain matters and define my learning direction more precisely. (Peer communication)
Teacher-student communication	B4: The students must first recognize the teacher before he can learn something from the teacher. If he does not recognize the teacher, they would just complete the task normally. If they want to study hard, they has to recognize the teacher very much, and this is what I’ve observed from my child. When my daughter first entered high school, both her academic performance and mental state suffered significantly. Later, a began discussing topics like promising university majors and future career paths with her. That teacher became her role model, and her own learning state has improved a lot more than before. (Teacher-student communication)
Recent expectations	B6: When it comes to his role model, he would feel that he should have such a good result in the future like his role model, and expect himself to grow up to be like his role model. (Expect short-term results)
Forward expectations	B2: Through the career courses, my daughter has determined that she wants to be a doctor. She has clear aspirations for her future, and I think this is a wonderful thing becuase she knows what she wants to do. In fact, many children do not know what they want to do, yet they do not know what they do not want to do either. How can they possibly excel in their studies if they just drift through each day? (Looking forward to future career)

Further analysis and induction of the 15 categories derived from open coding revealed the following results. Information collection and understanding of the environment were categorized under knowledge reserve. Self-assessment and self-reflection were categorized under cognitive internalization. Learning interest and learning motivation were categorized under power conversion. Target selection and making a plan were categorized under skill upgrading. Problem solving and logical thinking were categorized under skill transfer. Parent-adolescent communication, peer communication, and teacher-student communication were categorized under affective interaction. Recent expectations and forward expectations were categorized under positive expectations. To sum up, seven secondary dimensions were formed: knowledge reserve, cognitive internalization, power conversion, skill upgrading, skill transfer, affective interaction, and positive expectations. These seven secondary dimensions were then further analyzed and induced. We summarized knowledge reserve, cognitive internalization, and power conversion into cognition, skill upgrading and skill transfer into skill, and affective interaction and positive expectations into affection. This resulted in three main categories: cognition, skill, and emotion. The axial coding results are shown in Table 8.

**Table 8:** Axial coding results.

First-order dimension	Second-level dimension	Three-level dimension
Cognize	Knowledge reserve	Information collection
		Understanding of the environment
	Cognitive internalization	Self-assessment
		Self-reflection
	Power conversion	Learning interest
		Learning motivation
Skill	Skill upgrading	Target selection
		Making a plan
	Skill transfer	Problem solving
		Logical thinking
Affection	Affective interaction	Parent-adolescent communication
		Peer communication
		Teacher-student communication
	Positive expectation	Recent expectations
		Forward expectations

**Figure 1:**
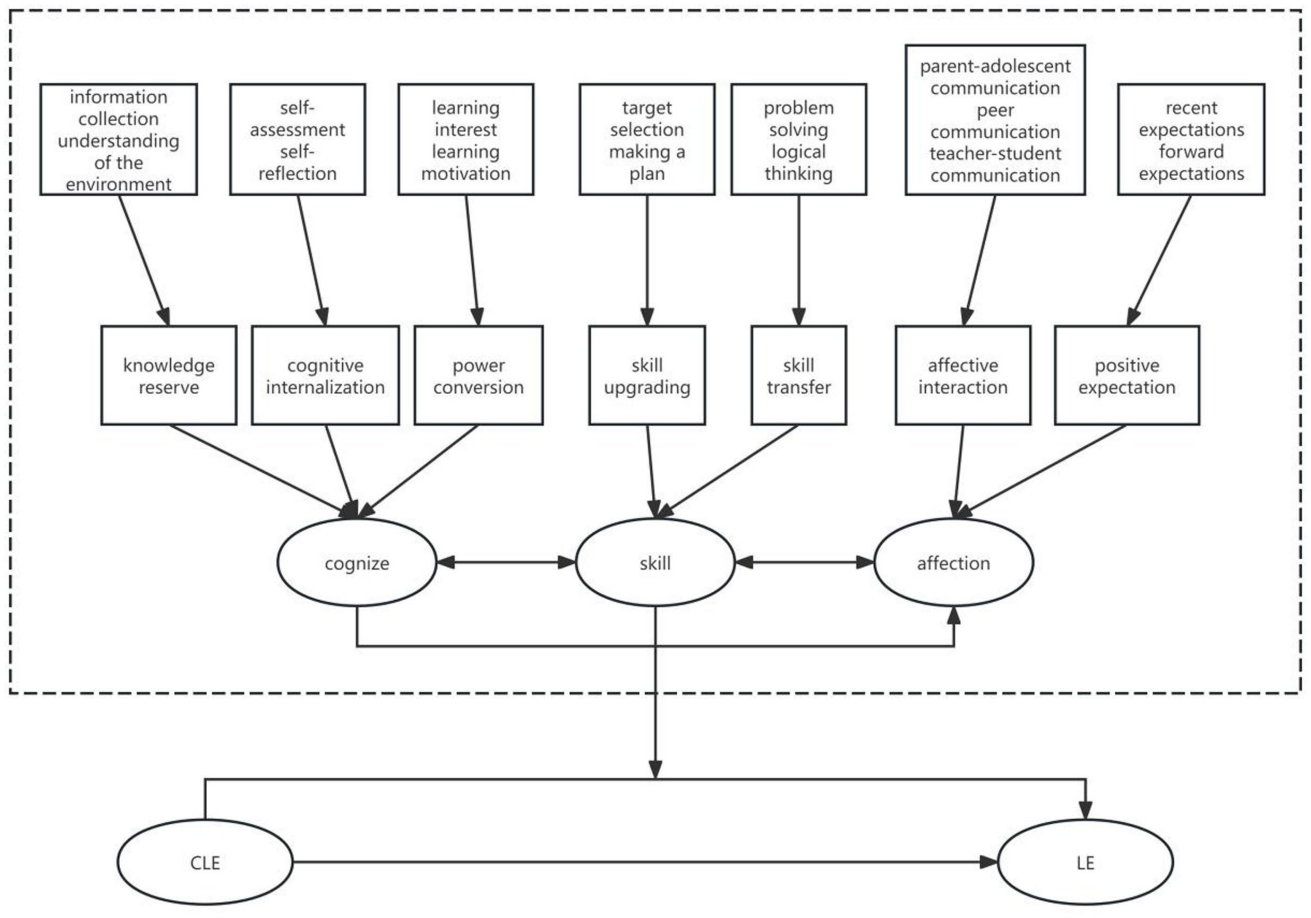
Mechanism model of the influence of career learning experience on high school students’ learning engagement; Career learning experience (CLE), Learning engagement (LE).

A comparative analysis of the three major categories derived from axial coding revealed that they all converge toward the core category of “how career learning experiences influence high school students’ learning engagement.” This ultimately established the core category of “the mechanism by which career learning experiences affect high school students’ learning engagement.” Based on this, a mechanism model of the influence of career learning experiences on high school students’ learning engagement is constructed, as shown in Figure 1. Specifically, career learning experiences may influence high school students’ learning engagement through three main categories: cognitive, skill, and affective. These categories also interact with each other, with each comprising distinct dimensions.

## 5 Conclusion and discussion

This study used a sequential explanatory mixed-methods design to examine the effects of career learning experience on high school students’ learning engagement and explore its underlying mechanisms. The results of quantitative analysis showed that career learning experience has a positive predictive effect on Chinese high school students’ learning engagement. Verbal persuasion, negative physiological and affective states, positive physiological and affective states, vicarious learning, and personal mastery experience can significantly predict learning engagement, too. This result validates the first research hypothesis of this study.

Firstly, verbal persuasion, affirmation from a trusted individual of one’s successful performance of one’s successful performance during career activities or encouragement to engage in career activities, has a positive impact on high school students’ learning engagement. In this study, when high school students participate in career activities, they often receive both behavioral and emotional support. In terms of behavioral support, parents or teachers may proactively assist students by collecting career information and providing platforms for them to have career experiences, thereby offering high school students more opportunities to understand and experience various careers. Regarding emotional support, career activities can prompt high school students to shift their perceptions of career-related activities, thereby stimulating positive learning emotions and enhancing their engagement in learning (Xiong et al., 2021; Perry et al., 2010). This finding aligns with Sağkal and Sönmez (2021) study, which argued that the support from significant others included behavioral support and emotional support in some activities, such as helping children experience activities in different career areas.

Secondly, positive physiological and affective states, defined as positive emotional states experienced during career activities, exert a significant positive influence on high school students’ learning engagement. Conversely, negative physiological and affective states, the negative emotional states experienced during career activities, exert a significant negative influence on learning engagement. In this study, high school students’ physiological responses during career-related activities influence their career self-efficacy, which in turn affects their learning engagement. This finding corroborates Bandura (1999) research, which proposed that physiological responses, or physiological arousal, can have an impact on individual self-efficacy. Furthermore, this result validates Connell (1990) Self-System Processing Theory, which posited that students’ self-efficacy directly and significantly positively affected the level of learning engagement. The finding also echoes research by Caprara et al. (2022), indicating that self-efficacy has a significant positive effect on student learning engagement.

Thirdly, vicarious learning, which occurs when individuals acquire complex behaviors by observing others’ career choices and outcomes, particularly those of role models, has a positive influence on high school students’ learning engagement. This study reveals that when high school students witness individuals with similar backgrounds or role models achieving success in career planning, they develop aspirations for such outcomes, thereby enhancing learning motivation and increasing learning engagement. This is consistent with Myers (2018) research, which found that vicarious learning occurs when individuals observe others’ experiences and derive meaning from them. It can promote individuals’ knowledge growth, skill enhancement, interpersonal relationship improvement, and heightened motivation (Myers, 2018).

Fourthly, mastery experiences, defined as the successful experiences individuals gain through direct participation in career activities, impact high school students’ learning engagement positively. This study found that successful experiences gained through participation in career activities enhance high school students’ career efficacy, learning abilities, thinking skills, and comprehensive competencies, thereby elevating their overall learning engagement levels. This supports findings from Blotnicky et al. (2018), indicating that experiential career activities not only strengthen students’ career efficacy but also facilitate their integration of knowledge and action.

The results of qualitative analysis suggested that career learning experiences may influence the level of Chinese high school students’ learning engagement through cognition, skill, and affection. This research finding supplements the influence mechanism of career learning experience on learning performance in the SCCT theory.

Firstly, the cognition dimension includes three aspects: knowledge reserve, cognitive internalization, and power conversion. Knowledge reserve includes information collection and understanding of the environment. The successful or vicarious experiences that high school students gain through personal participation in certain career fields enable them to learn more effective ways to collect information about their career fields, gain a better understanding of their intended majors and careers, increase their knowledge reserve of the external career world (or how to understand it), and ultimately enhance their learning goals and learning motivation, thereby promoting their level of learning engagement (Kim and Choi, 2021). Cognitive internalization includes self-assessment and self-reflection. High school students internalize the vicarious experiences gained from personal participation in certain career field activities or from observing career role models, which enables them to conduct objective self-evaluation and reflection, enhance self-cognition, and improve learning engagement (Zhou et al., 2021). Power conversion includes learning interest and learning motivation. The improvement of the level of career learning experience has a positive impact on the selection of subjects and majors of high school students, making them more clear about the purpose of major selection, stimulating the interest and motivation of learning, clarifying the purpose and significance of learning, and promoting the improvement of the level of learning engagement (Hsieh, 2014).

Secondly, the skill dimension includes skill upgrading and skill transfer. Skill upgrading includes target selection and making a plan. High school students learn how to choose subjects and majors and how to make effective plans from the successful experience or vicarious experience of participating in certain career field. In this process, they exercise their ability to make independent choices and plans and improve their learning efficiency. This makes high school students have more energy and psychological adaptability in learning and promotes the improvement of the level of learning engagement (Docherty et al., 2018). Skill transfer involves problem-solving and logical thinking. The successful experience or alternative experience of participating in a career field is conducive to cultivating high school students’ positive outlook on life, improving their social responsibility, innovative spirit, and practical ability, and promoting the improvement of comprehensive ability (Nurtanto et al., 2020). In this way, when facing other problems outside the career field, high school students can maintain a positive attitude, solve problems with scientific and rigorous methods, and persevere even when encountering difficulties in learning, thus enhancing their learning engagement (Veas et al., 2015).

Thirdly, the affection dimension includes affective interaction and positive expectation. Affective interaction includes parent-adolescent communication, peer communication, and teacher-student communication. In the face of career decisions such as major selection, many high school students experience tension, anxiety, and other emotions, and they communicate more with parents, peers, and teachers to seek help and relieve pressure. Positive parent–child, peer, and teacher-student relationships have a positive predictive effect on high school students’ learning engagement level (Banks and Smyth, 2021). Positive expectations include recent expectations and forward expectations. Through personal involvement in career field activities or the experience of watching career role models, high school students have expectations about their learning outcomes and future majors and careers. Outcome expectation is an individual’s prediction of the outcome of their own behavior, and positive expectation is conducive to the improvement of high school students’ learning engagement level (Pinquart and Ebeling, 2020).

## 6 Recommendations

Career planning education for high school students in China is still in its infancy. Many schools, especially those in underdeveloped areas, lack related teachers and suitable venues, making it difficult to organize career learning activities for their students. Therefore, based on the research findings, this study offers some suggestions for governments, schools, and parents. At the governmental level, it is imperative to fully mobilize resources from all sectors of society and integrate regional career education resources to establish comprehensive career learning platforms. Furthermore, the government should collaborate with social support to develop career experience centers, encourage these centers to organize career exploration and vocational experience activities for students, and strive to build a social support system for career education. This will furnish high school students with opportunities to engage in experiential learning activities related to their careers, thereby gaining more successful experiences across diverse professional fields and enhancing their overall career efficacy. At the school level, career planning courses can be introduced to guide students to attach importance to career learning. Additionally, schools can provide professional development for existing subject teachers to design career education-infused courses and integrate their career learning experiences into teaching practices. Such innovative course designs and teaching integration can foster students’ positive attitudes toward academic subjects, stimulate their learning interest and motivation, and thereby enhance their learning engagement levels. This can also promote students’ self-awareness and comprehension of related subjects, assisting them in synthesizing career learning experiences and selecting appropriate college entrance exam subjects. At the parental level they should actively encourage their children to explore career paths and provide behavioral and emotional support for their career exploration. On one hand, parents should proactively assist their children in gathering relevant career information and providing platforms for them to experience certain careers, among other things. Such behavioral support can provide more opportunities for students to understand and experience various professions and help them clarify their subject selections and academic goals, thereby fostering a sense of purpose in their studies. On the other hand, parents should convey trust in their children and provide timely positive feedback on their career exploration activities. Such emotional support can enhance high school students’ academic engagement and ultimately promote their learning engagement.

## 7 Limitations and future prospects

Our study still has some limitations. First, in terms of quantitative research tools, two scales employed in this study are both Chinese translations of English-version instruments, which do not highlight the group characteristics of Chinese high school students. Therefore, the scales used in this study require further refinement in the aspect of cross-cultural adaptability. In the future, we should develop relevant scales within China to enhance the appropriateness of the tools. Second, this study employed convenience sampling from two schools in one city, which limits the generalizability of the findings to other regions or school types in China. Future research should replicate this study with a larger, more representative sample across multiple provinces. The qualitative phase included only nine student–parent dyads; although thematic saturation was achieved, the exploratory nature of these findings should be acknowledged, and further qualitative work with diverse populations is needed to deepen the mechanistic understanding. Due to the exploratory nature of the qualitative phase and the limited sample size, the thematic analysis should be interpreted as providing illustrative insights rather than definitive mechanistic evidence. The coding process followed established thematic analysis procedures, but formal inter-coder reliability was not calculated given the primary aim of generating hypotheses for future research. In terms of qualitative research conclusions, the author’s limited experience in qualitative research resulted in findings that did not achieve the anticipated depth when constructing the mechanism model of influence of career learning experiences on learning engagement. In the future, we will make efforts to deepen the understanding and application of qualitative research methods, to yield more profound research discoveries. Furthermore, given the opportunity, we plan to validate the findings of this study through questionnaire surveys, curriculum interventions, and other methods.

## Data Availability

The raw data supporting the conclusions of this article will be made available by the authors, without undue reservation.
